# Performance evaluation and TiGRA-based multi-response optimization of sustainable fly ash-slag-based one-part alkali-activated concrete mix design

**DOI:** 10.1038/s41598-025-34746-1

**Published:** 2026-01-07

**Authors:** Prabhu Gurunathappa Sheelavantar, Poornachandra Pandit, Shreelaxmi Prashant

**Affiliations:** Manipal Institute of Technology, Manipal Academy of Higher Education, Manipal, Karnataka India

**Keywords:** One-part alkali-activated concrete, Multi-response optimization, Taguchi-integrated-GRA approach, Mechanical properties, Permeability properties, Civil engineering, Composites

## Abstract

The need for sustainable, practical alternatives to Portland cement and two-part alkali-activated systems has led to the development of one-part alkali-activated concrete (OPAAC), which efficiently reuses industrial by-products like fly ash and ground granulated blast furnace slag (GGBS). This study evaluates the fresh (workability) and hardened properties (compressive, tensile, and flexural strengths) of FA-GGBS-based OPAAC, along with durability indicators including sorptivity and chloride ion permeability. A performance-based multi-response optimization using a Taguchi L_9_ array and Grey Relational Analysis (GRA) is adopted to optimize the binder ratio (FA: GGBS), water-to-binder ratio (w/b), and activator-to-binder ratio (A/b). SEM and XRD analyses indicate that the porous, N-A-S-H–dominated matrix observed in FA-rich mixes gradually evolves into a denser C-A-S-H/N-C-A-S-H gel network as the GGBS content increases, correlating with improved strength and durability. The optimal OPAAC mix, achieving an M40 grade concrete, is obtained at a 70:30 binder ratio, a water-to-binder ratio (w/b) of 0.35, and an activator-to-binder (A/b) ratio of 14%. This work directly supports global sustainability efforts by promoting low-carbon materials, resource efficiency, and environmentally responsible construction, aligning with SDGs 9, 11, 12 and 13.

## Introduction

 Concrete made from Portland cement continues to dominate the construction industry, providing reliable durability for various structures. The cement industry plays a substantial role in the release of greenhouse gases^1^, releasing about a ton of CO_2_ for every ton of cement produced, accounting for approximately 7% of global CO_2_ emissions^2,3^. Moreover, Ordinary Portland Cement (OPC) production depletes natural resources since they are essential ingredients for its manufacture^4,5^. This reliance on traditional cement poses sustainability challenges due to its significant environmental issues^6^.

Researchers are exploring alternative materials such as ground granulated blast furnace slag (GGBS), fly ash (FA), metakaolin, and rice husk ash to mitigate these environmental impacts. These materials, derived from industrial and agricultural by-products, offer potential substitutes for conventional cement. However, challenges such as disposal and landfilling also need to be addressed^7^, to fully harness their benefits in reducing carbon footprints and promoting sustainability in concrete applications^8^.

Reinforced concrete (RC) structures face durability issues primarily due to rebar corrosion^9^. In addition to decreasing the service life, corrosion also incurs significant economic costs in maintenance and repairs^10–12^. In India, corrosion-related expenses account for 3–4% of the national GDP annually^13^. Understanding the root causes of rebar corrosion and implementing effective preventive measures are essential to improve the durability and longevity of RC structures.

Factors contributing to concrete deterioration include moisture content, temperature variations, pH reduction, and permeability^14^. The penetration of aggressive fluids containing Cl^-^ ions and CO_2_ into the concrete accelerates degradation, highlighting the importance of producing denser concrete to slow this process^15^. Integrating supplementary cementitious materials (SCMs) known for their finer particle sizes, such as FA and GGBS, can substantially decrease permeability while enhancing the overall durability of concrete^16,17^.

**Fig. 1:**
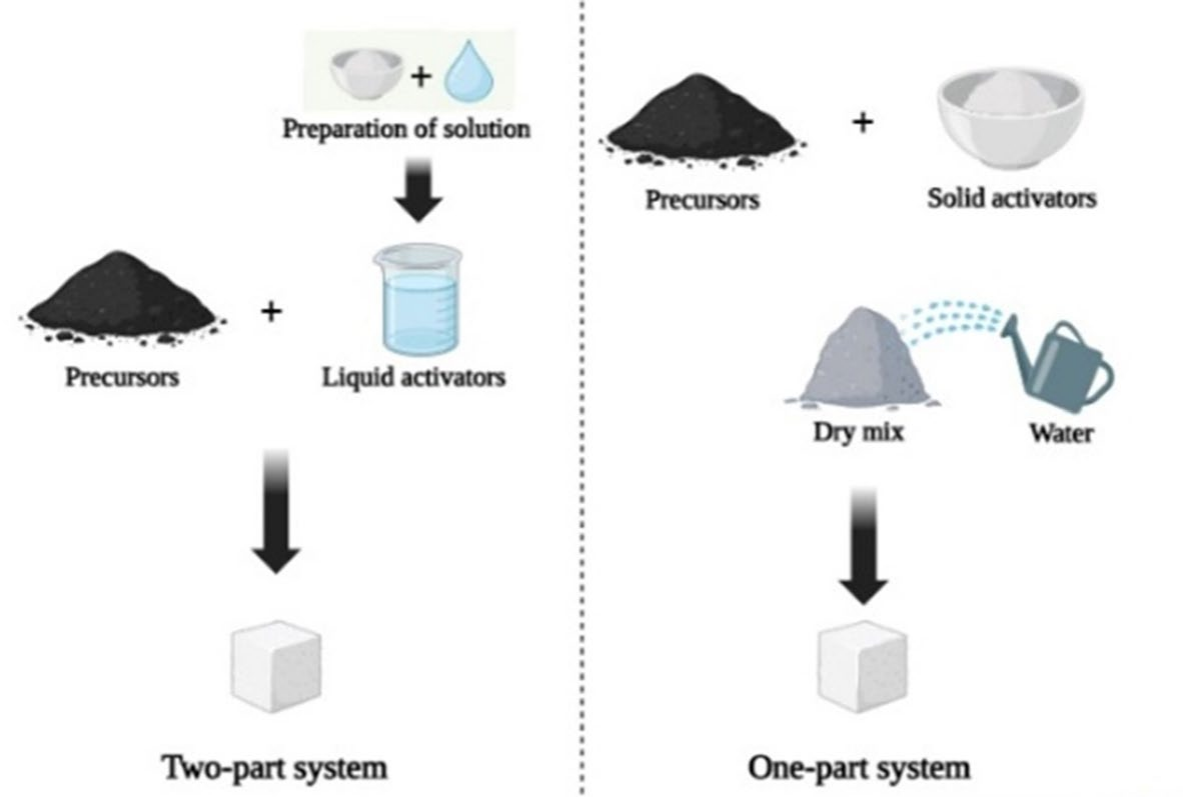
Two-part v/s One-part alkali-activation system.

Innovations in materials science have led to the emergence of alkali-activated concrete (AAC) and composite materials as potential replacements for traditional concrete binders^18^. Alkali-activated materials, synthesized from industrial and agricultural by-products, are activated using alkaline activators to develop properties comparable to OPC-based binders. This technology, evolving towards a “one-part alkali-activation” or “just add water” approach (as shown in Fig. 1), eliminates the need for preparing complex activator solutions (usually alkaline hydroxide and silicates), thus reducing energy-intensive processes and enhancing application feasibility^19,20^. Additionally, AACs demonstrate a significant reduction in embodied energy, typically in the range of 40–50%, while their equivalent embodied CO_2_ values are 70–75% lower compared to OPC concrete^21,22^. The carbon footprint of OPAACs is 60% lower than that of OPC-based concrete and 30% lower than that of two-part AAC^19^.

The performance of OPAAC is influenced by the precursor composition, water-to-binder (w/b) ratio, activators, and their proportions, and curing regimes. The widespread implementation of OPAAC is constrained by the current gap in establishing a reliable standardized mix design procedure^23^. The formulation of mix design methodology for OPAAC continues to pose challenges owing to its intricate dependence on multiple interacting variables^24^. Key variables such as binder ratio, w/b ratio, activator-to-binder (A/b) ratio, and sodium silicate to sodium hydroxide (Na_2_SiO_3_/NaOH) ratio play a critical role in determining the properties of OPAAC^25^. Achieving optimal mix proportions requires extensive trial mixes and a comprehensive understanding of each variable’s role in determining the mechanical and durability characteristics of AAC.

AAC uses industrial and agricultural by-products as precursors, with FA and GGBS being the most widely used binders. The use of FA alone promotes N-A-S-H gel development, associated with prolonged setting times and requires heat curing^26^, posing challenges for practical applications^27^. Adding GGBS helps to form C-A-S-H gel, regulating setting time and enhancing strength without heat^5^. However, using GGBS in excess can lead to rapid setting and reduced workability, which may affect casting and compaction. Therefore, a higher proportion of FA is maintained to improve workability and long-term durability, while a limited amount of GGBS is added to balance the setting time and early strength gain. Activation is done using sodium hydroxide (NaOH), sodium metasilicate (Na_2_SiO_3_), or both, with combined use accelerating reactions more effectively than single activators^28^.

Extensive research has explored the impact of significant factors on the workability, strength, and durability characteristics of AAC^29–33^. However, research on the multi-objective optimization of FA-GGBS-based AAC targeting both strength and durability remains limited. Addressing this gap offers significant potential to transform these industrial by-products into sustainable construction materials. The Taguchi method has been employed to reduce experimental trials and identify optimal mix proportions^34–36^, but it mainly supports single-response optimization. To overcome this, Grey Relational Analysis (GRA), a widely adopted technique in mechanical engineering disciplines^37–41^, is increasingly being applied in construction materials research^42–50^.

This study employs the Taguchi approach to efficiently minimize the number of experimental trials while systematically analyzing the impact of each factor and its levels on the performance of OPAAC, including workability, mechanical strength, and durability properties. GRA is employed to optimize the mix design for multiple performance responses of structural-grade OPAAC. The results demonstrate the effectiveness of statistical approaches in optimizing various concrete properties.

## Materials and methods

### Materials

**Fig. 2:**
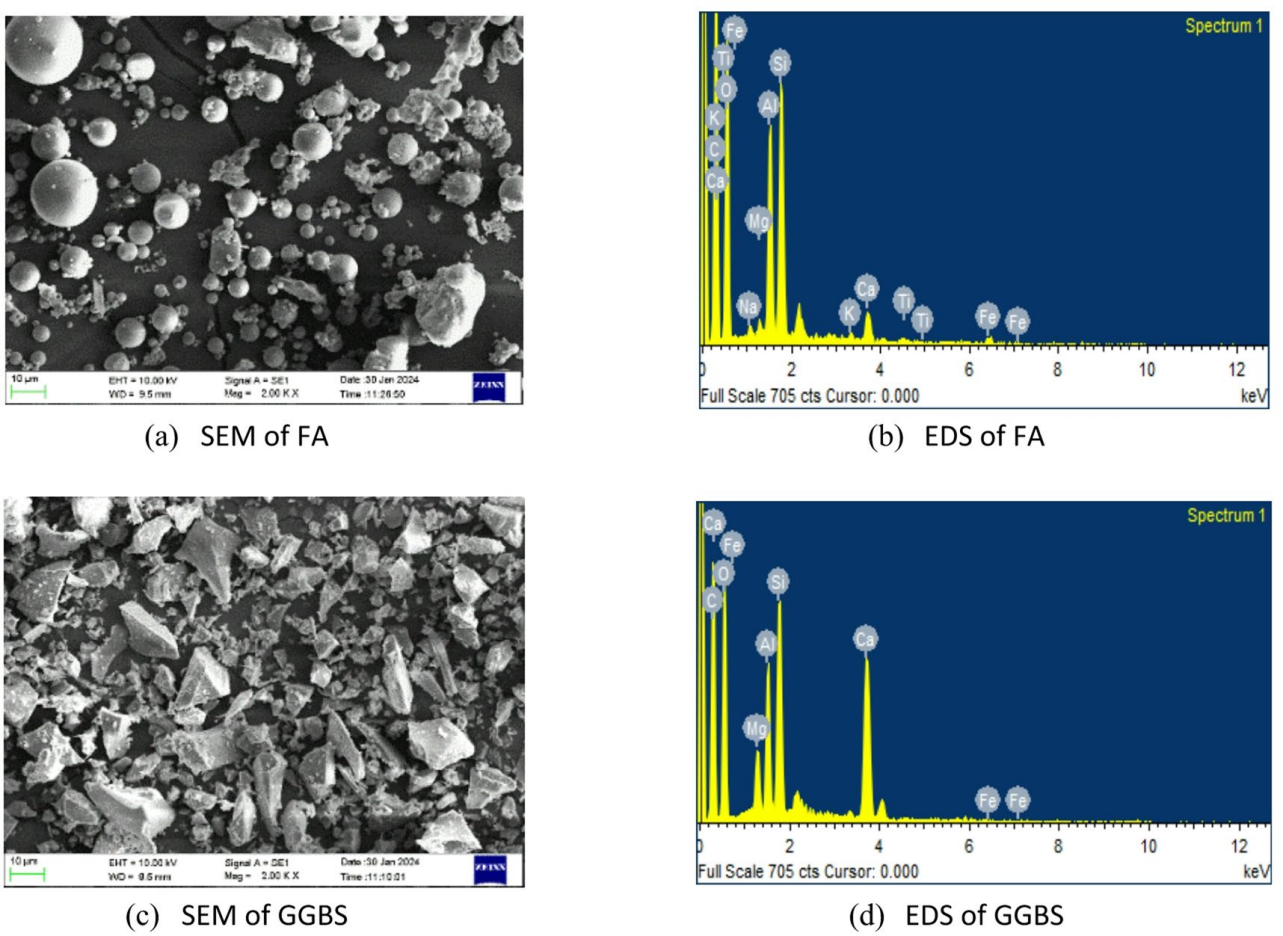
SEM and EDS of precursors.

**Fig. 3:**
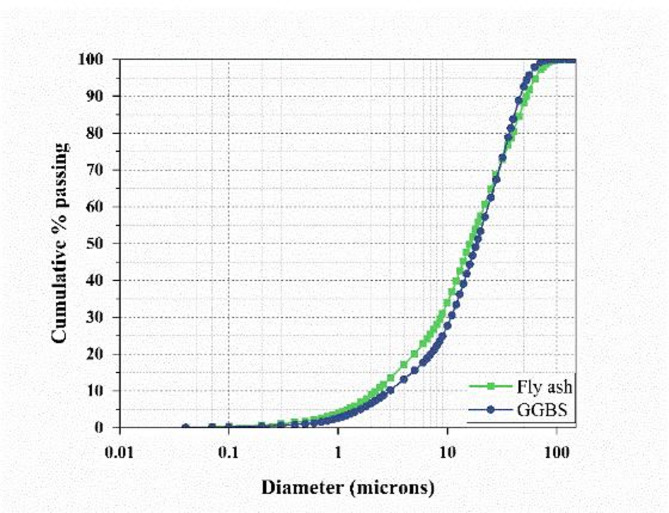
Particle size distribution of precursors.

Class F fly ash, procured from the Udupi Thermal Power Plant (UPCL) and complying with IS 3812 (Part 1):2013^51^ serves as a primary precursor along with GGBS supplied by JSW, Hosapete, Karnataka, conforming to IS 16714:2018^52^, serves as the secondary binder. Figure 2 presents the SEM and EDS analysis of the precursors. The particle size distribution of FA and GGBS was measured using a laser diffraction analyzer. The dried samples were dispersed and analyzed to obtain their characteristic particle sizes. The D_10_, D_50_, and D_90_ values for FA were 2.23, 16.07, and 52.93 μm, respectively, while those for GGBS were 2.94, 18.43, and 46.44 μm. Figure 3 presents particle size distribution of precursors. The chemical composition of FA and GGBS was determined using X-ray fluorescence (XRF). The fine samples passing 75 μm were placed in the instrument chamber, and elemental oxides were quantified based on calibrated fluorescence spectra. Table 1 presents the chemical composition analysis obtained through the XRF test.

**Table 1:** Chemical composition of precursors.

	SiO_2_	Al_2_O_3_	CaO	MgO	K_2_O	Na_2_O	TiO_2_	SO_3_	MnO_2_	Fe_2_O_3_	LOI
**FA**	46.70	26.30	2.92	1.85	0.61	0.42	1.89	1.56	0.20	15.77	1.78
**GGBS**	37.3	16.6	34.7	6.87	0.63	0.31	0.82	1.35	0.96	0.37	0.09

Sodium hydroxide (NaOH) and sodium metasilicate (Na_2_SiO_3_) are the solid activators, with NaOH at 98% purity and Na_2_SiO_3_ featuring a Na_2_O/SiO_2_ ratio of 1:1 ± 0.05. Locally sourced aggregates include 20 mm downsize coarse aggregate (specific gravity 2.65, water absorption 0.837%) and 4.75 mm downsize river sand (specific gravity 2.62, water absorption 0.6%), both conforming to IS 383:2016^53^.

### Treatment combination and orthogonal array

In the present study, key factors, including the binder ratio, w/b ratio, and A/b ratio, were investigated at three different levels. The binder ratio, defined as the proportion of FA to GGBS, was selected based on existing literature^46^, demonstrating that a 90:10 binder ratio can achieve M40-grade AAC. Accordingly, the proportion of FA substituted with GGBS was varied from a minimum of 10% to a maximum of 30%, as higher GGBS content accelerates the setting and induces premature cracking^54,55^. In addition to the rapid setting and cracking issues already noted, high GGBS content in OPAAC also increases sensitivity to activator dosage and accelerates the reaction of high calcium in GGBS^56,57^. It accelerates workability loss, which may pose challenges during mixing and placement^58^. These factors underscore the need for careful control of temperature, mixing sequence, and curing practices when using GGBS-rich formulations in field applications. The w/b ratios ranging from 0.35 to 0.40 were determined through preliminary trials to ensure compatibility with all binder ratios. Similarly, the A/b ratio was selected based on both literature review^59^ and experimental observations, with segregation noted in mixtures exceeding an A/b ratio of 0.14, and thus, the upper limit was restricted accordingly. Further, variables such as curing regime play a significant role in the performance of OPAAC. The present study focuses on formulating a FA-GGBS-based OPAAC mix that is curable at ambient temperature, suitable for large-scale construction. Therefore, the curing condition is maintained the same for all the mixes. Table 2 shows the selected treatment combinations.

**Table 2:** Treatment Combination.

Factors	Levels		
	1	2	3
**Binder ratio (FA: GGBS)**	90:10	80:20	70:30
**w/b**	0.350	0.375	0.400
**A/b (%)**	10%	12%	14%

Experimental trials with all combinations are laborious and time-intensive. The Taguchi method was implemented using an L_9_ orthogonal array to optimize the experimental design, substantially reducing the required trials and systematically organizing nine experimental runs to evaluate the variables at their designated levels. Table 3 presents the combination of factors and their levels for the trial mixes. Levels 1 to 3 denote the minimum to maximum values for each factor. Binder ratio (Factor 1) uses GGBS at 10% (Level 1) to 30% (Level 3) by total binder mass, so FA ranges from 70 to 90%. The w/b ratio (Factor 2) governs workability, beginning at 0.350 and increasing in 2.5% increments by binder weight. A/b ratio (Factor 3) and activator composition influence polymerization. A Na_2_SiO_3_/NaOH ratio of 2 optimizes silicate dosage^62^, and total A/b ratios of 10%, 12%, and 14% are evaluated.

**Table 3:** Experimental trial mixes with factors and levels.

Trial ID	Binder ratio	w/b	A/b
T1	90:10	0.350	10
T2	90:10	0.375	12
T3	90:10	0.400	14
T4	80:20	0.350	12
T5	80:20	0.375	14
T6	80:20	0.400	10
T7	70:30	0.350	14
T8	70:30	0.375	10
T9	70:30	0.400	12

### Mix design methodology

**Fig. 4:**
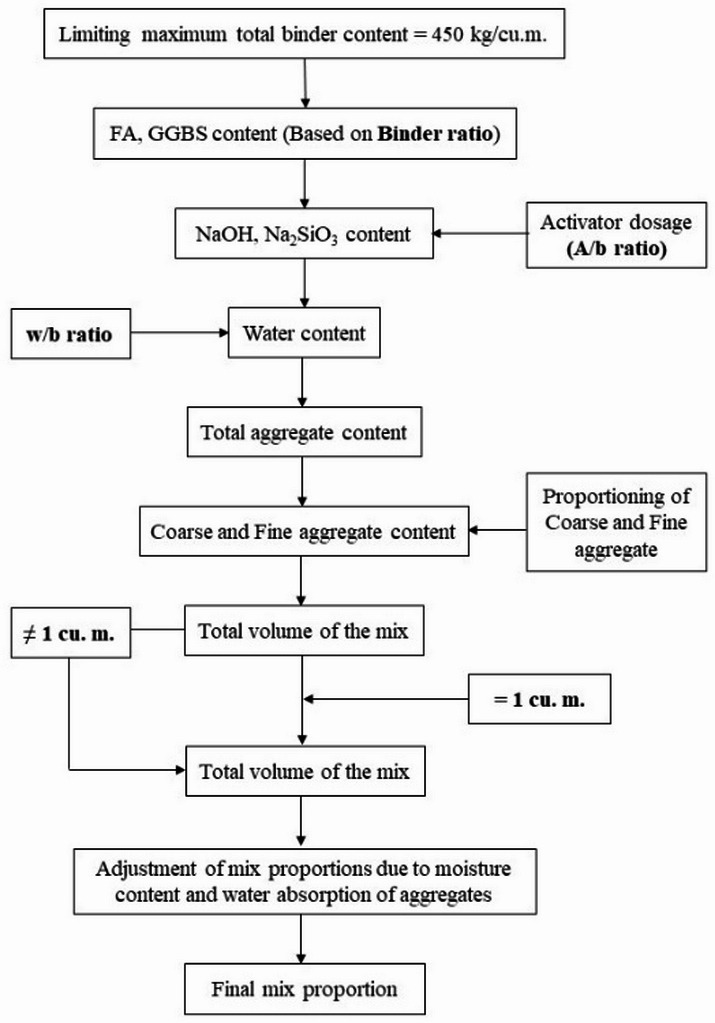
Flowchart of mix design methodology.

The present study adopts a mix design methodology, as shown in Fig. 4, which follows the absolute volume method. The absolute volume method was adopted for its simplicity and practical applicability, associated with precise volumetric proportioning of all constituents. Its key advantage over other methods is that it accounts for the true volume contribution of each material, enabling consistent and reproducible OPAAC mixes. The mix proportions are tabulated in Table 4.

**Table 4:** Mix proportions in kg/m^3^.

Mix ID	T1	T2	T3	T4	T5	T6	T7	T8	T9
**FA**	405	405	405	360	360	360	315	315	315
**GGBS**	45	45	45	90	90	90	135	135	135
**NaOH**	15	18	21	18	21	15	21	15	18
**Na**_**2**_ **SiO**_**3**_	30	36	42	36	42	30	42	30	36
**CA**	1118	1089	1059	1116	1087	1083	1115	1111	1082
**Sand**	474	461	449	473	461	459	472	471	458
**Water**	180	191	203	180	191	203	180	191	203

### Mixing and specimen Preparation

One‑part and two‑part alkali‑activated concretes differ mainly in activator incorporation. In OPAAC, precursors, solid activators, and aggregates are dry‑mixed before water is added to achieve homogeneity; this process parallels conventional cement mixing and is thus more suitable for large‑scale use. Two‑part AAC, by contrast, is favored in research for its precise activator control.

In one‑part mixing, aggregates are first dry‑mixed for 2–3 min, followed by adding precursors and solid activators and mixing for 4–5 min, then gradually adding water until a uniform mix is achieved. The fresh mix undergoes slump testing, then is cast into 150 mm cubes, 150 × 300 mm cylinders, and 100 × 100 × 500 mm prisms for mechanical testing, and 100 × 50 mm cylinders for sorptivity and chloride ion permeability, with each mold consolidated on a vibrating table. The specimens are cured for 28 days under ambient temperature with average room temperatures of 32 °C to 35 °C.

### Testing of specimens

 The slump cone test is performed to evaluate the workability of each mix as per IS 1199 (Part 2): 2018^63^ guidelines. Compressive strength, split-tensile, and flexural strength tests are conducted for all mixes as per IS 516 (Part 1/Sect. 1): 2021^64^. Additionally, permeability characteristics, including sorptivity and chloride ion permeability, are evaluated following ASTM C 1585-20^65^ and ASTM C 1202-22^66^, respectively.

### Analysis methods

The study utilizes the Taguchi method, employing the means and signal-to-noise (S/N) ratios to evaluate the influence of key factors on the properties of OPAAC. Additionally, surface plots are analyzed to illustrate the interaction between two independent variables and the dependent variable. Microstructural analyses are conducted using scanning electron microscopy (SEM) and X-ray diffraction (XRD) to complement and validate the mechanical and durability results, providing insights into the morphology and phase composition of the developed concrete mixes.

For SEM analysis, small fragments obtained from the crushed compression-tested specimens were cleaned, oven-dried at 60 °C, and sputter-coated with a thin layer of gold to ensure conductivity and minimize charging effects. The imaging was carried out using a ZEISS EVO18 Special Edition microscope equipped with a Tungsten/LaB₆ electron source and a backscattered electron (BSE) detector. Micrographs were captured at various magnifications to observe the reaction products, matrix densification, pore structure, and presence of unreacted particles.

To identify the crystalline phases present, XRD analysis was performed using a fifth-generation Rigaku MiniFlex 600 diffractometer, which is capable of scanning over a wide 2θ range (5°–130°). Prior to testing, the samples were crushed and sieved through a 75 micron mesh to obtain a uniform powder suitable for diffraction analysis. The instrument parameters included a step size of 2°, a scanning range of 0°–90° 2θ, and a scan speed of 2°/min.

## Results and discussion

### Workability

The w/b ratio significantly influences concrete workability, with increased ratios enhancing workability to an optimal limit, beyond which further increase may result in segregation and bleeding^67^. Most OPAAC mixes have achieved a pumpable concrete slump of 150 mm. A slump of approximately 150 mm is suitable for pumpable concrete, offering sufficient flowability for efficient placement and ease of casting in reinforced sections. However, its performance may vary under field conditions, as factors such as temperature, moisture, and transport time can influence workability, requiring proper handling and control measures.

**Fig. 5:**
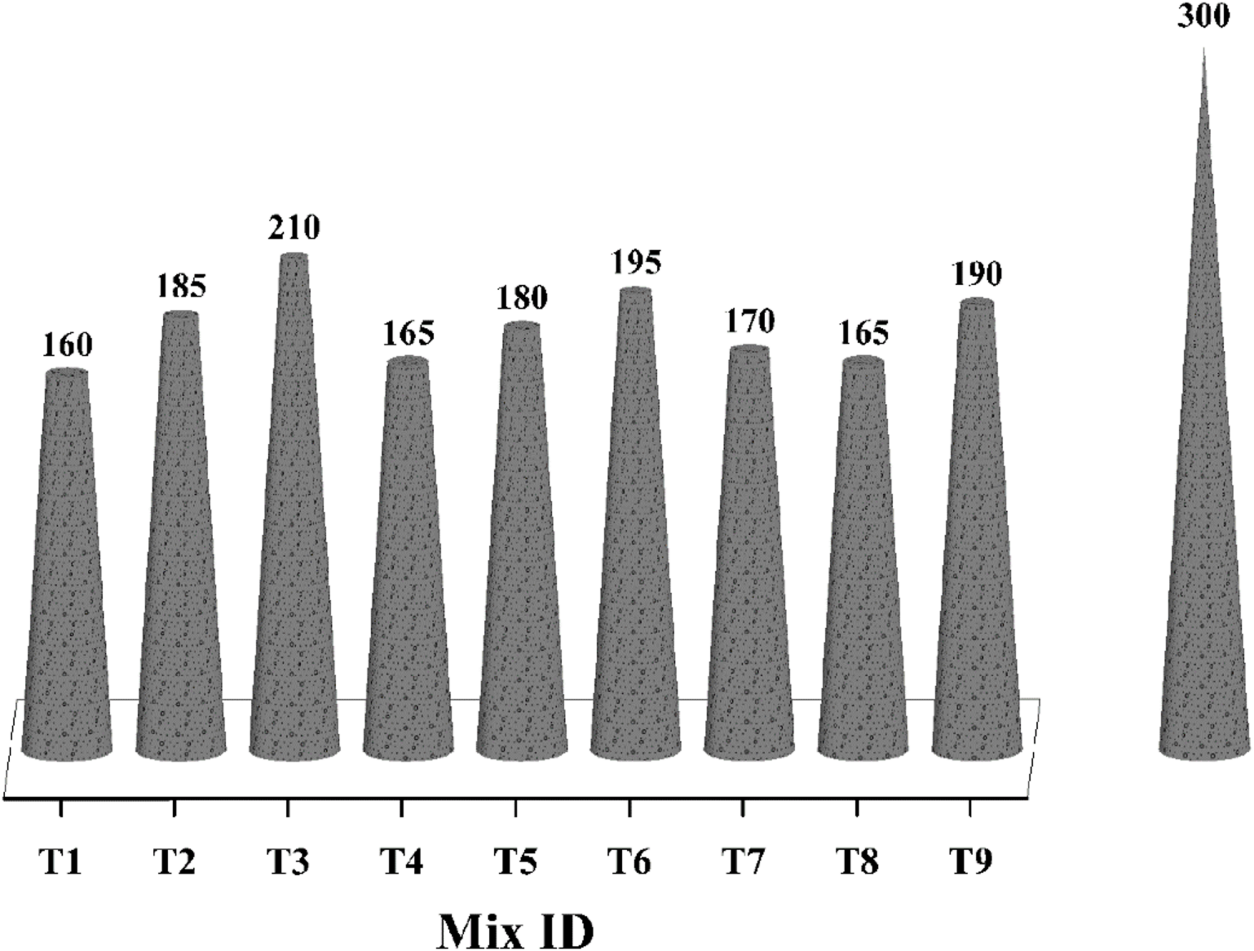
Slump test results.

The directly proportional relationship between water content in the mix and slump can be seen in Fig. 5. Besides, the higher proportion of GGBS significantly impacts the workability of concrete due to early setting and irregular particle shape, causing internal friction^1^. Further, the A/b ratio also has a considerable effect on workability^68^, and the combined impact of the w/b ratio and activator dosage has increased slump values to a greater extent in the OPAAC mixes.

**Fig. 6:**
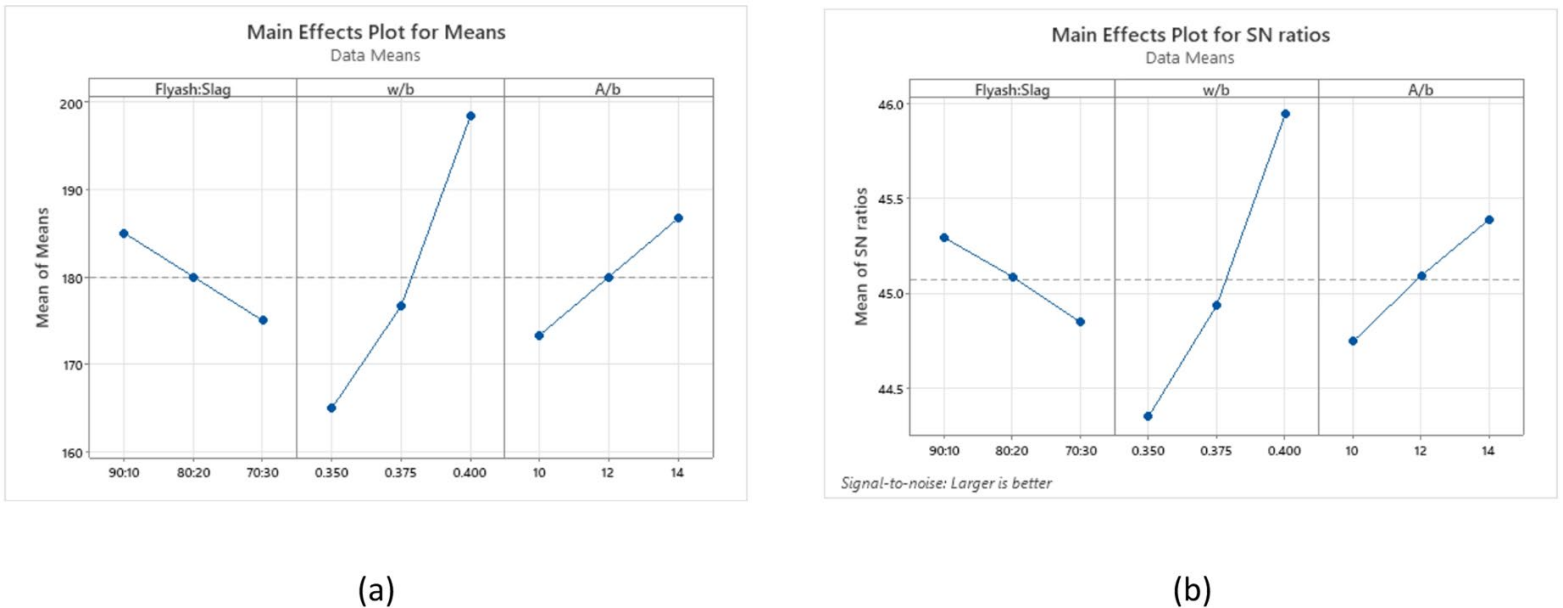
Main effects plot for (**a**) means (**b**) S/N ratios of slump.

The evaluation of slump values employs S/N ratios, emphasizing the “larger is better” criterion for facilitating the ease of handling fresh concrete. Figure 6(a) displays the mean of the slump values, and corresponding S/N ratios are presented in Fig. 6(b). The most significant factor influencing workability is the w/b ratio, followed by the binder ratio and A/b ratio. The maximum slump can be achieved through the combination of a 90:10 binder ratio, a 0.4 w/b ratio, and a 14% A/b ratio.

**Fig. 7:**
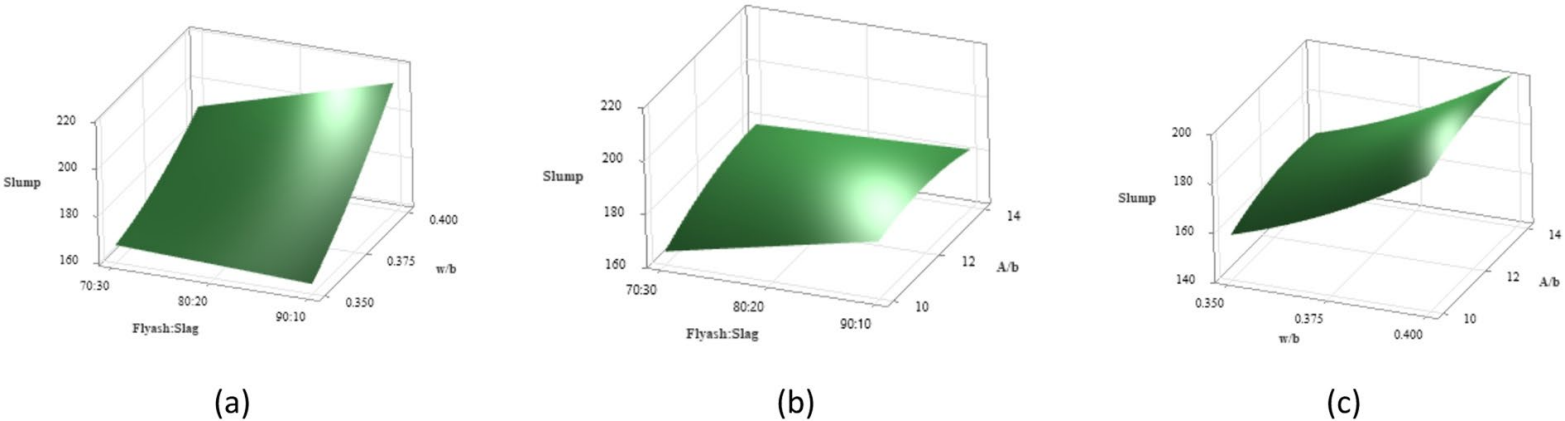
3D Surface plots for slump (**a**) Slump vs. Binder ratio, w/b. (**b**) Slump vs. Binder ratio, A/b (**c**) Slump vs. w/b, A/b.

The 3D surface plots depicted in Fig. 7 illustrate the combined effects of influencing factors on slump values. In Fig. 7(a), it is evident that an increase in the w/b ratio leads to higher slump values when the binder ratio remains constant. Among the different binder ratios examined, the mix with a 70:30 binder ratio exhibited the lowest slump, primarily due to its higher content of GGBS, which tends to increase interparticle friction. Conversely, the 80:20 ratio possessed a higher slump compared to 70:30, and the highest slump was observed with the 90:10 ratio at a w/b ratio of 0.40, likely due to its higher FA content, owing to its spherical particle shape, which reduces interparticle friction, resulting in enhanced workability characteristics^69^.

Figure 7(b) demonstrates that increasing the A/b ratio while keeping the binder ratio fixed results in higher slump values. Higher activator dosages increase the concentration of dissolved silicate anions, which act as plasticizers, improving workability without altering the water or binder content^29^. The mix with 80:20 binder ratio showed improved workability compared to 70:30, while the 90:10 ratio with a 14% A/b ratio achieved the maximum slump because of higher FA content. This improvement is attributed to spherical FA particles reducing internal friction and the higher A/b ratio facilitating better particle lubrication and reduced viscosity, enhancing workability^70^.

Figure 7(c) demonstrates the combined influence of w/b ratio and A/b ratio as surface plot. Maintaining a constant w/b ratio in OPAAC and increasing the A/b ratio leads to higher slump values, as the additional dissolved silicate anions act as dispersing agents, enhancing workability^71^. Lower slumps were observed at lower w/b ratios, suggesting insufficient water content that forms a more viscous mix. Slump values increased moderately with higher w/b ratios. The combination of a 0.40 w/b ratio and a 14% A/b ratio yielded the highest slump. This combination resulted in a less viscous mix, where the higher w/b ratio and A/b ratio contributed to improved workability.

### Compressive strength

**Fig. 8:**
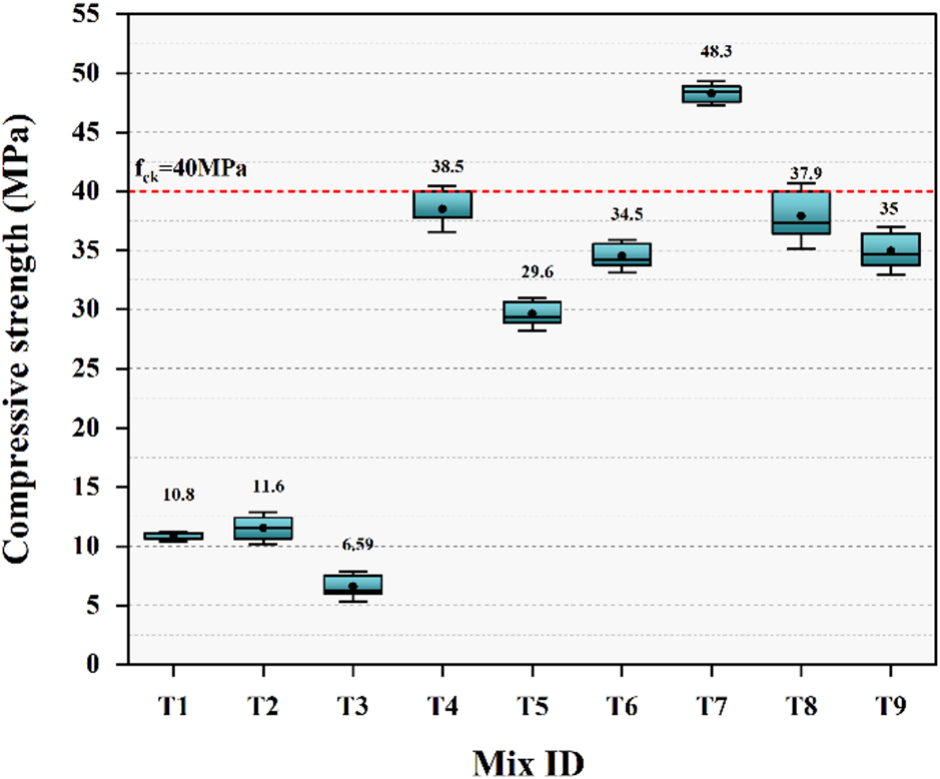
Compressive strength results.

The compressive strength of all nine mixes was evaluated after 28 days of ambient curing, and the corresponding results are illustrated in Fig. 8. Mix T7 demonstrated the highest compressive strength of 48.3 MPa, while T3 demonstrated the lowest strength of 6.59 MPa.

Figure 8 illustrates that the mixes with higher GGBS content exhibit increased compressive strength due to the presence of Ca-rich binding phases with denser microstructure compared with the more porous structure of fly-ash-rich systems^72^. Mix T7 exhibits peak strength among the mixes studied due to the highest GGBS, enhancing C-A-S-H cross-linking. Conversely, the low strength in mixes such as T3 is primarily due to their unfavorable combination of high FA content and a higher w/b ratio, which limits gel formation and results in a porous matrix. These findings are supported by microstructural analysis discussed in the later sections. Further, higher A/b ratios marginally reduce strength by inducing microcracking^73^, but the primary influence remains GGBS content on strength development.

Based on the observed performance trends, mix combinations yielding excessively low strength such as those with high FA content and higher w/b ratios are considered impractical for structural applications and are therefore not recommended for further development.

**Fig. 9:**
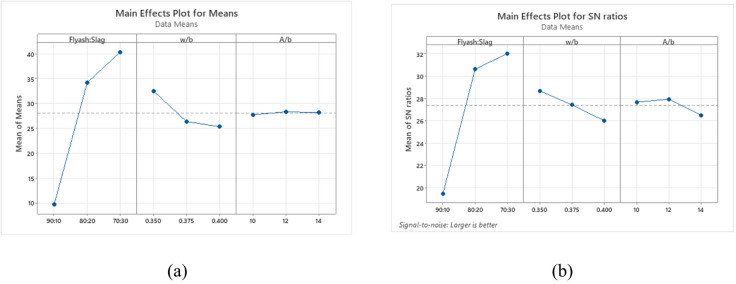
Main effects plot for (**a**) Means (**b**) S/N ratios of compressive strength.

The compressive strength analysis follows the principle that a higher S/N ratio corresponds to improved performance. According to Fig. 9, the binder ratio plays the most critical role, followed by the w/b ratio and A/b ratio. Figure 9(a) and 9(b) demonstrating optimal conditions observed at a binder ratio of 70:30, a 0.350 w/b ratio, and a 12% A/b ratio.

**Fig. 10:**
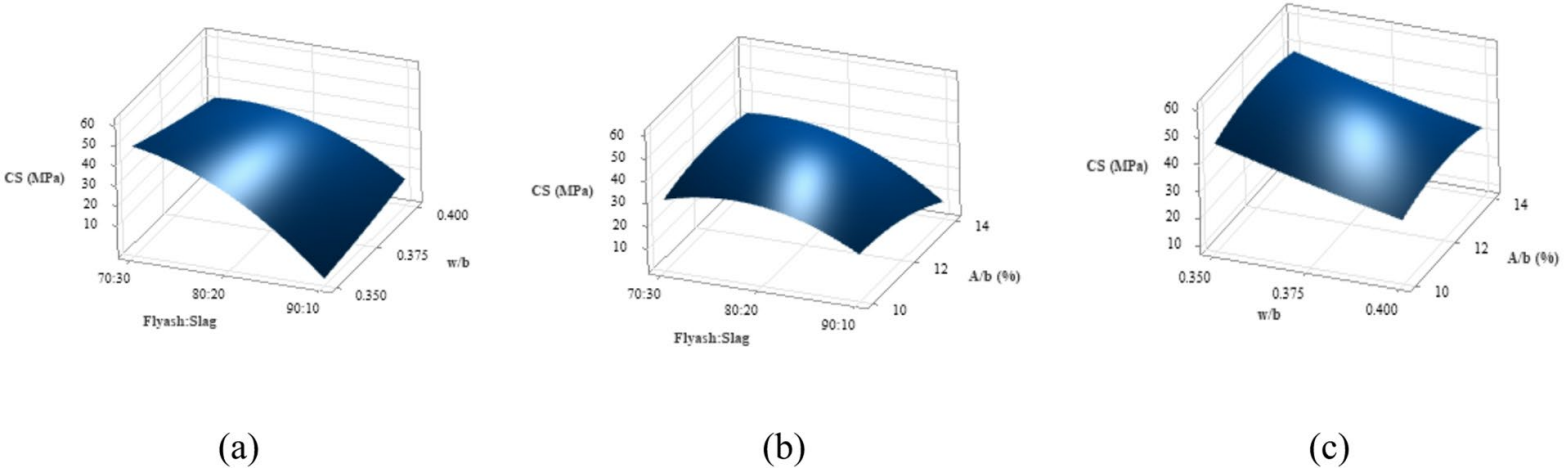
3D Surface plots for compressive strength (**a**) Compressive strength vs. Binder ratio, w/b (**b**) Compressive strength vs. Binder ratio, A/b (**c**) Compressive strength vs. w/b, A/b.

Figure 10 illustrates 3D surface plots that capture the combined influence of key factors on compressive strength. In Fig. 10(a), when the binder ratio is kept constant, increasing the w/b ratio consistently results in decreased compressive strength. This is because excess water leads to higher porosity and dilution of the binder matrix, compromising strength development^71^. Among the evaluated binder ratios, the 70:30 binder ratio exhibited the highest compressive strength primarily due to the higher GGBS content, which promotes the formation of dense calcium-alumino-silicate-hydrate (C-A-S-H) gels that enhance matrix strength^58^. Although the 80:20 mix demonstrated slightly lower strength, the lowest strength was recorded for the 90:10 binder ratio at a w/b of 0.350. The decline in strength at this composition may be attributed to the combined effect of the lower w/b ratio and higher FA content. The reduced availability of free water for precursor dissolution, together with the inherently slow-reacting nature of fly ash, can limit overall binder reactivity and lead to a less compact microstructure, as further supported by the microstructural analysis presented later.

Figure 10(b) demonstrates that increasing the A/b ratio while maintaining a constant binder ratio leads to only marginal increases in compressive strength. The main contributor to strength remains the binder composition. The 70:30 binder ratio consistently outperforms others due to higher GGBS content. The dissolution of Ca²⁺ ions, accelerating the formation of C–A–S–H gel, which fills voids more effectively and produces a denser, more cohesive microstructure compared with FA-rich mixes and improves microstructural densification. Furthermore, increasing the activator dosage facilitates more extensive geopolymerization, further contributing to strength development^74^. The observed synergistic effect indicates that optimizing both the binder ratio and activator dosage is crucial for achieving superior compressive strength in alkali-activated systems.

Figure 10(c) presents the interaction between A/b ratio and w/b ratio on compressive strength. At a constant w/b ratio, increasing the A/b ratio slightly improves strength by enhancing geopolymerization efficiency within an optimal range^68^. The w/b ratio of 0.350 delivered the highest strength, primarily due to its lower water content, which reduces porosity and enhances matrix density^71^. The 0.375 w/b ratio produced moderately lower strength, while the 0.45 w/b ratio with a 10% A/b ratio gave the lowest strength. Utilizing a lower w/b ratio and a higher activator dosage can maximize the compressive strength. A denser gel network and reduced porosity, facilitated by optimal activator content and controlled water addition, are critical for achieving structural grade OPAAC.

### Tensile strength

**Fig. 11:**
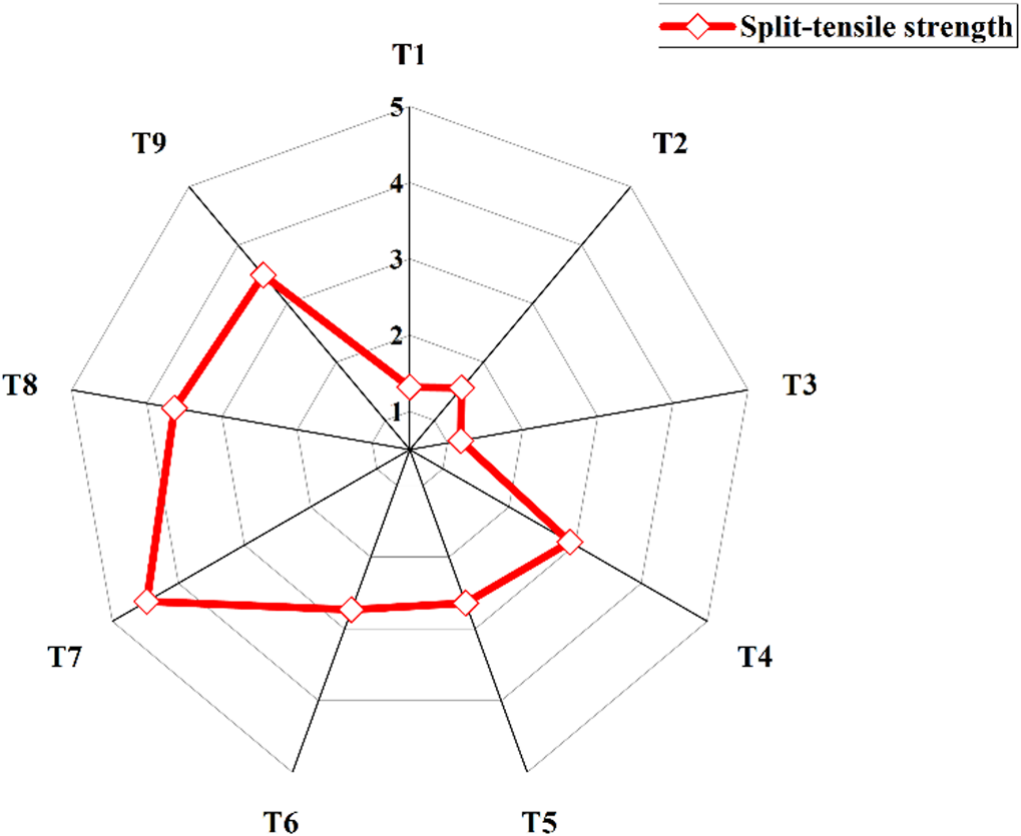
Split-tensile strength results.

After subjecting all nine mixes to ambient temperature curing for 28 days, the split-tensile strength test was conducted. Figure 11 presents the average split-tensile strength values with three specimens prepared for each mix. T7 exhibited the highest split-tensile strength, reaching 4.48 MPa, while T3 showed the lowest strength at 1.18 MPa. It can be observed that only T7 satisfied the empirical relationship established between compressive and split-tensile strengths (f_ct_ = 0.7*√f_ck_), as proposed for cement concrete^75^.

**Fig. 12:**
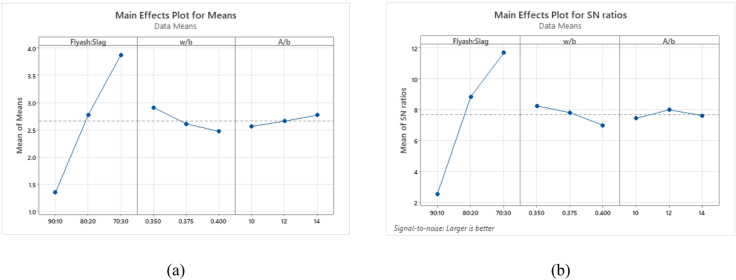
Main effects plot for (**a**) Means (**b**) S/N ratios of split-tensile strength.

Figure 11 illustrates that mixes T7 and T3 exhibited the highest and the lowest split-tensile strength among the mixes studied. This variation is mainly attributed to the GGBS content, as the binder ratio is the most influential factor affecting split-tensile strength. These results directly corelate with compressive strength results and microstructural developments in the matrix of the mixes. Figure 12(a) presents the mean split-tensile strength responses, while Fig. 12(b) illustrates the S/N ratios computed considering “Larger-the-better” criterion. Like compressive strength, the hierarchical significance of factors follows binder ratio, w/b ratio, and A/b ratio. The 70:30 binder ratio, w/b ratio of 0.350, and 14% A/b ratio yielded the highest mean strengths, while 12% A/b showed the highest S/N ratio.

**Fig. 13:**
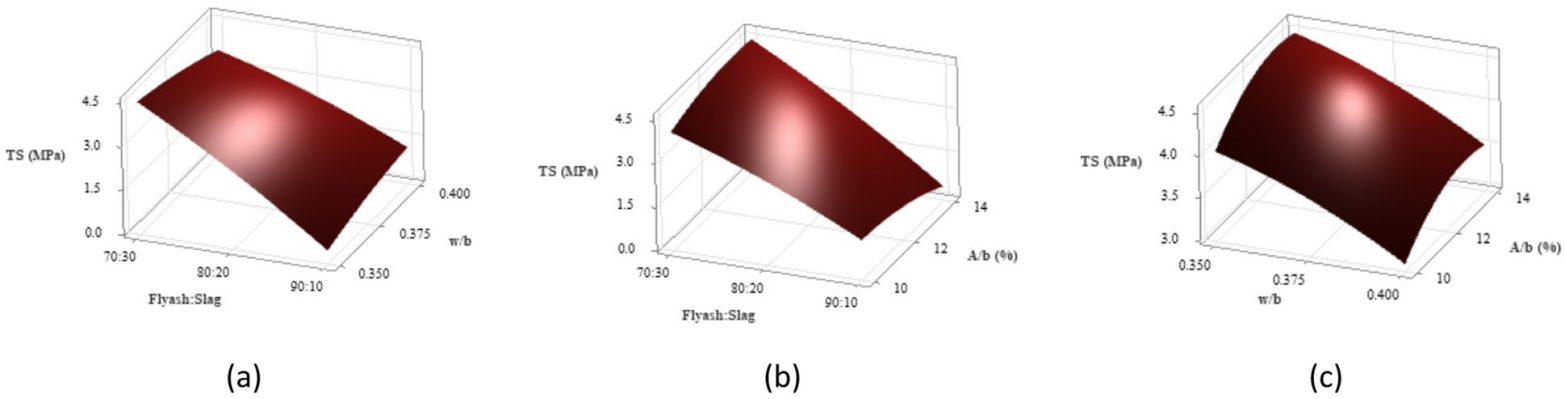
3D Surface plots for split-tensile strength (**a**) Split-tensile strength vs. Binder ratio, w/b (**b**) Split-tensile strength vs. Binder ratio, A/b (**c**) Split-tensile strength vs. w/b, A/b.

Figure 13 presents 3D surface plots illustrating the quantitative effects of key variables on split-tensile strength. In Fig. 13(a), an increase in the w/b ratio at a constant binder ratio leads to a reduction in split-tensile strength, reflecting a trend similar to that observed in compressive strength. The 70:30 binder ratio exhibited the highest split-tensile strength among the investigated binder ratios. This is attributed to its superior compressive strength and higher GGBS content, which improves matrix cohesion and enhances overall strength development. The 80:20 binder ratio exhibited slightly lower tensile strength, while the 90:10 mix at a 0.350 w/b ratio showed the lowest value. This reduction likely stems from inadequate water and a higher FA proportion, which limits binder reactivity and results in a porous, weaker microstructure that reduces both compressive and tensile strength.

Figure 13(b) shows that increasing the A/b ratio at a constant binder ratio typically yields a modest rise in split-tensile strength, consistent with compressive strength trends. The 70:30 binder ratio consistently achieved the highest tensile strength due to greater GGBS content, resulting in a denser and cohesive matrix. The 80:20 ratio provided moderate strength, while the 90:10 mix yielded the lowest values across all A/b ratios, attributed to reduced GGBS and weakened matrix cohesion. The binder ratio remains the most influential factor, with the 70:30 mix performing best due to its optimal reactivity and matrix density. The decline in tensile strength in mixes with high FA and excessive activator content is likely due to disruption of optimal chemical reactions and a less compact microstructure, affecting overall performance^76^.

Figure 13(c) depicts the combined impact of w/b ratio and A/b ratio on split-tensile strength. At a fixed w/b ratio, increasing the A/b ratio generally enhances tensile strength, aligning with compressive strength patterns. The highest tensile strength was observed at a 0.350 w/b ratio, as lower water content fosters a denser, more cohesive matrix. Upon increasing the w/b ratio to 0.375 and subsequently to 0.400, a gradual strength decline occurred, with the lowest strength at a 0.400 w/b and 10% A/b combination. This drop is due to excess water and limited activator content, which dilute reactants and disrupt matrix continuity, producing a porous structure and weakening mechanical performance^77^.

### Flexural strength

**Fig. 14:**
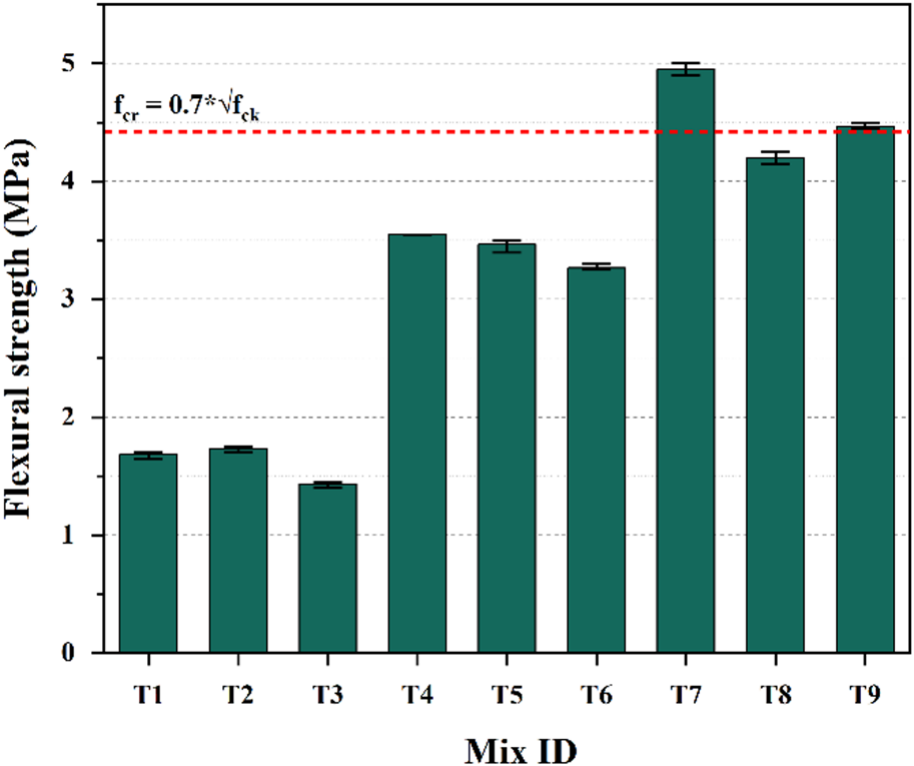
Flexural strength results.

The flexural strength test results depicted in Fig. 14 present the average flexural strength values for each of the mixes after 28 days of curing, with three specimens tested per mix. Mix T7 exhibited the highest flexural strength among all mixes, achieving 4.95 MPa. In contrast, T3 exhibited the lowest strength, recording value of 1.43 MPa. Like split-tensile strength, only T7 and T9 met the empirical relationship between flexural strength and compressive strength (where f_cr_ = 0.7*√f_ck_), as specified for conventional cement-based concrete^75^.

Only mixes T7 and T9 met the empirical tensile and flexural strength relationships, reflecting their balanced proportions and well-developed reaction products. The deviations observed in the other mixes are attributed to inadequate gel formation and higher porosity, indicating limited stability of the mixes. These findings suggest that such formulations are unreliable for structural-grade OPAAC and should be avoided in practice.

**Fig. 15:**
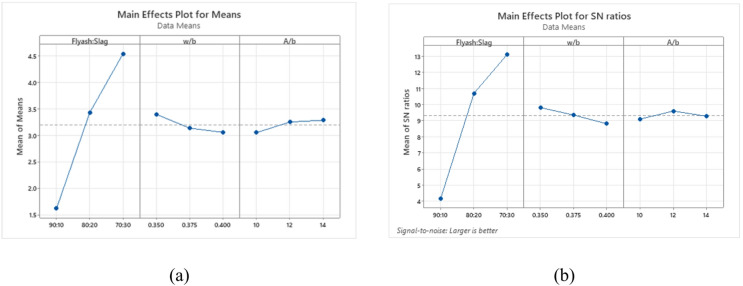
Main effects plot for (**a**) Means (**b**) S/N ratios of flexural strength.

The analysis of flexural strength outcomes highlights the significant impacts of key factors across different levels. Figure 15(a) presents average flexural strength findings, while Fig. 15(b) shows the corresponding S/N ratios using the “Larger-the-Better” criterion. The hierarchical influence of factors ranks the binder ratio first, followed by the w/b ratio, and then the A/b ratio. Specifically, a 70:30 binder ratio achieved the highest mean flexural strength, with a w/b ratio of 0.350, which similarly delivered optimal results. Additionally, an A/b ratio of 14% yielded the highest mean flexural strength, while 12% exhibited the largest S/N ratio.

**Fig. 16:**
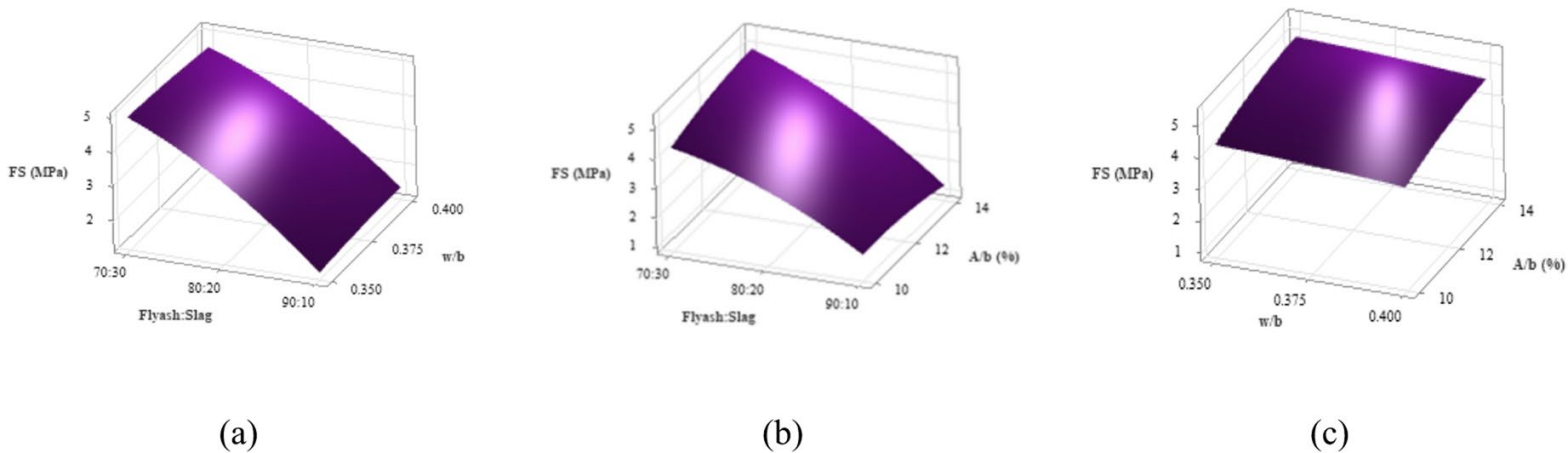
3D Surface plots for flexural strength (**a**) Flexural strength vs. Binder ratio, w/b. (**b**) Flexural strength vs. Binder ratio, A/b (**c**) Flexural strength vs. w/b, A/b.

Figure 16 displays 3D surface plots that illustrate the quantitative influence of multiple variables on flexural strength. In Fig. 16(a), increasing the w/b ratio while keeping the binder ratio constant causes only a slight decrease in flexural strength. The 70:30 binder ratio consistently yields the highest flexural strength among the binder combinations. This is attributed to its higher GGBS content, which enhances binder reactivity and leads to a denser, more cohesive matrix. The 80:20 binder ratio produces slightly lower strength, while the 90:10 mix at a 0.350 w/b ratio shows the lowest strength, likely due to insufficient water and high FA content weakening the paste-aggregate bond and reducing resistance to bending, a trend observed in both compressive and split-tensile strength reduction with similar conditions.

Figure 16(b) shows that increasing the A/b ratio at a fixed binder ratio improves flexural strength. The 70:30 binder ratio again exhibits superior flexural strength due to optimal GGBS content promoting better geopolymerization and matrix densification. In contrast, the 90:10 mix at 10% A/b ratio shows the lowest strength, likely from high FA content and low activator dosage disrupting the formation of reaction products.

Figure 16(c) presents the combined effect of w/b ratio and A/b ratio on flexural strength. At constant w/b ratios, increasing the A/b ratio results in a slight improvement in flexural strength. However, mixes with fixed A/b ratios exhibit similar strength values, indicating a negligible interaction effect between w/b ratio and A/b ratio.

### Sorptivity

**Fig. 17:**
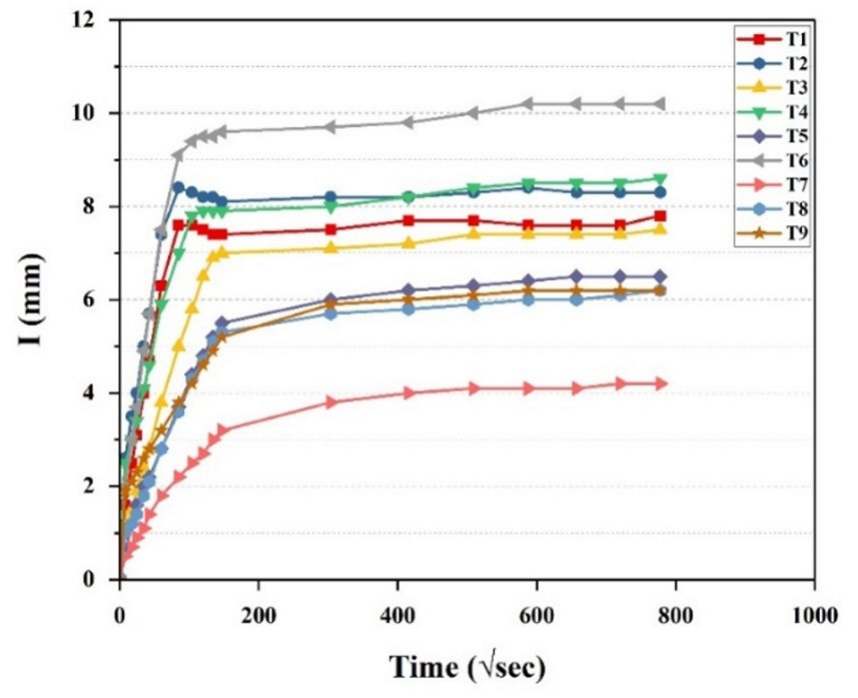
Sorptivity test results.

All nine trial mixes were evaluated for sorptivity in accordance with ASTM C1585^65^. The results indicated a consistent trend of increasing sorption over time across all mixes; however, the rate of sorption varied among them, as shown in Fig. 17. Mix T7 exhibited the most favorable performance, recording the lowest sorptivity of 0.0042 mm, whereas T6 demonstrated the highest sorptivity of 0.0102 mm.

**Fig. 18:**
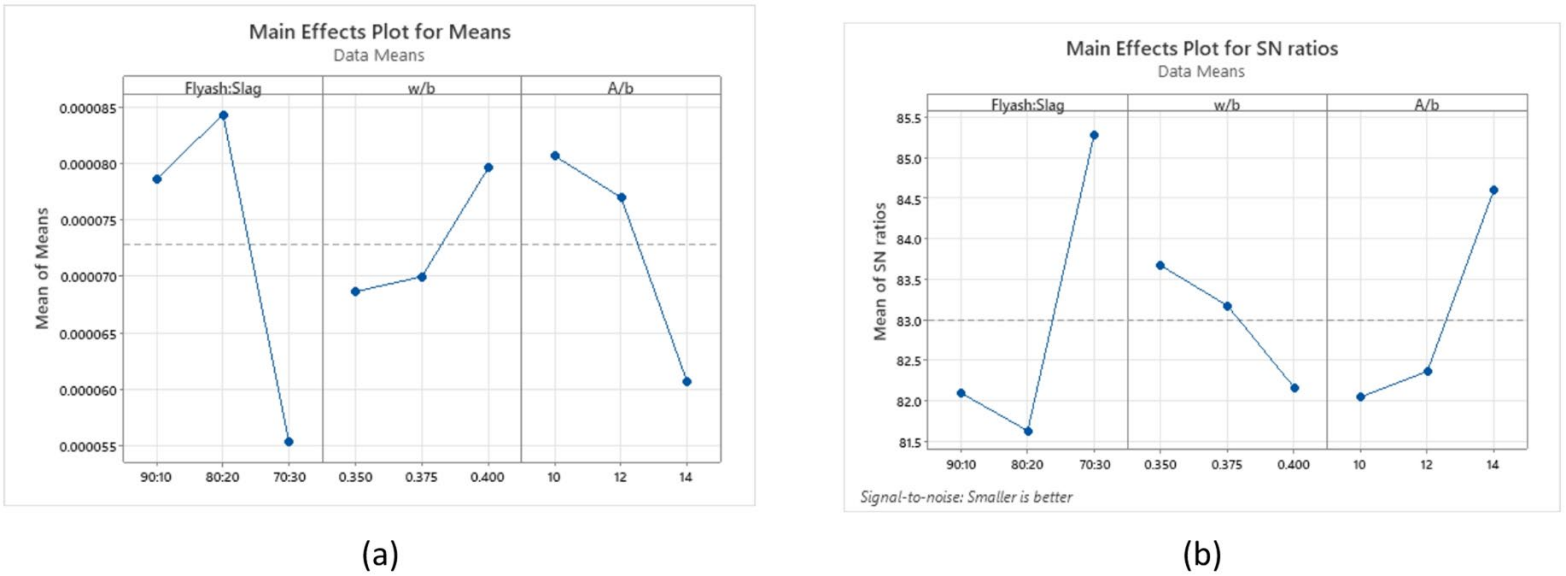
Main effects for (**a**) Means (**b**) S/N ratios of sorptivity.

Sorptivity analysis was conducted using Signal-to-Noise (S/N) ratios, where lower S/N values indicate improved performance, as higher sorptivity is detrimental to concrete durability. Figure 18(a) presents the average sorptivity values, while Fig. 18(b) illustrates the corresponding S/N ratios. Among the evaluated parameters, the binder ratio was found to be the key factor governing sorptivity, followed by the A/b ratio, and subsequently the w/b ratio, indicating their relative significance in descending order. The mix incorporating a 70:30 binder ratio, a 0.350 w/b ratio, and a 14% A/b ratio exhibited the lowest sorptivity values, suggesting enhanced durability performance.

**Fig. 19:**
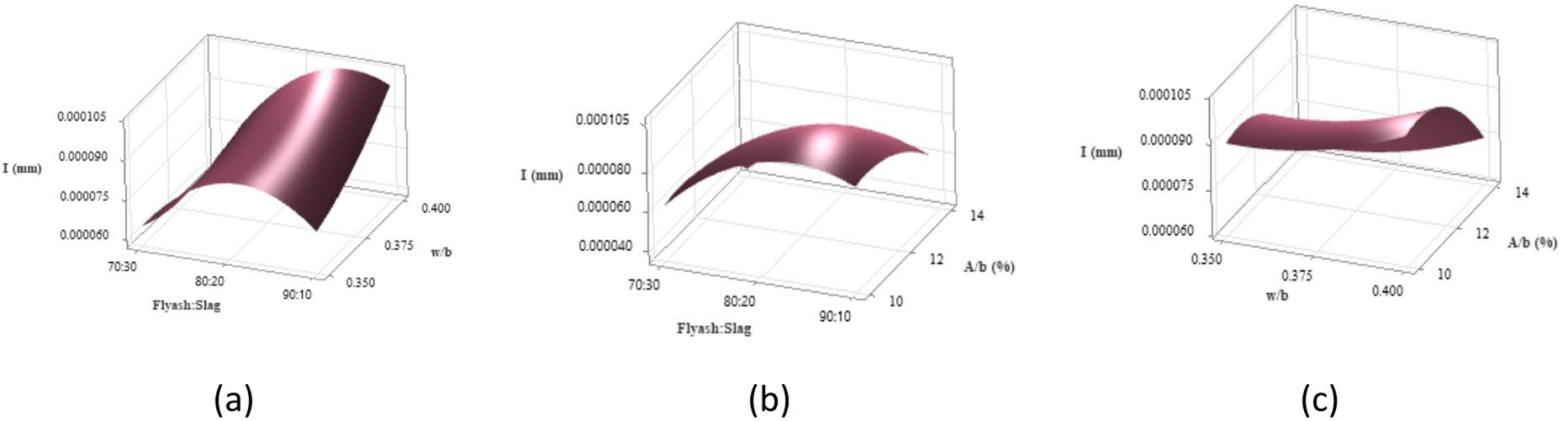
3D Surface plots for sorptivity (**a**) Sorptivity vs. Binder ratio, w/b. (**b**) Sorptivity vs. Binder ratio, A/b (**c**) Sorptivity vs. w/b, A/b.

Figure 19 illustrates 3D surface plots depicting the quantitative influence of key variables on sorptivity. In Fig. 19(a), the combined impact of the binder ratio, w/b ratio on sorptivity is observed. Higher w/b ratios result in increased capillary absorption due to the greater porosity in the concrete matrix. The 70:30 binder ratio demonstrated the most favorable performance among the tested binder ratios, exhibiting the lowest sorptivity corelating with enhanced compressive strength. This improvement is result of enhanced geopolymerization driven by the calcium content in GGBS, which promotes the formation of a denser microstructure through C-A-S-H gel development. This reduces the number and connectivity of pores^78^. On the other hand, the 80:20 binder ratio demonstrated the highest sorptivity, likely due to insufficient GGBS content that disrupted the gel network and led to the formation of larger capillary pores. Although the 90:10 mix performed better than 80:20, its limited GGBS content still resulted in a less continuous pore structure, allowing greater water penetration compared to the 70:30 binder ratio. These results emphasize the importance of optimal GGBS content in achieving a durable and compact microstructure.

Figure 19(b) shows that increasing the A/b ratio slightly reduces sorptivity, as higher activator concentrations enhance geopolymerization and result in a denser matrix. However, excessive activator dosages may increase porosity. The 70:30 binder ratio again achieved the lowest sorptivity due to the calcium-rich nature of GGBS, which encourages the development of C-A-S-H gels that refine pore structure and limit water absorption. This also correlates with its superior compressive strength, reflecting the effectiveness of combining optimal GGBS content with suitable activator levels. In contrast, the 80:20 mix showed higher sorptivity and the 90:10 ratio was recorded as the highest, as excessive FA content disrupted gel formation and created more interconnected pores. High FA levels with increased activator dosages can also lead to microcracks and discontinuous pores from w/b ratio imbalances, ultimately compromising strength and durability^76^.

Figure 19(c) presents the combined effects of w/b ratio and A/b ratio on sorptivity. At a fixed w/b ratio, increasing the A/b ratio led to a slight increase in sorptivity, indicating greater capillary absorption with higher activator content. The mix with a 0.350 w/b ratio displayed the lowest sorptivity, attributed to reduced water content, which contributed to a denser matrix and the highest compressive strength. As the w/b ratio increased to 0.375 and 0.400, sorptivity also rose, reflecting the adverse impact of excess water on pore connectivity and permeability. The highest sorptivity was observed at the combination of a 0.400 w/b ratio and 12% A/b ratio, where the elevated water and activator content likely created a more open pore network, thereby lowering overall compactness and reducing both durability and strength.

### Chloride permeability by RCPT

**Fig. 20:**
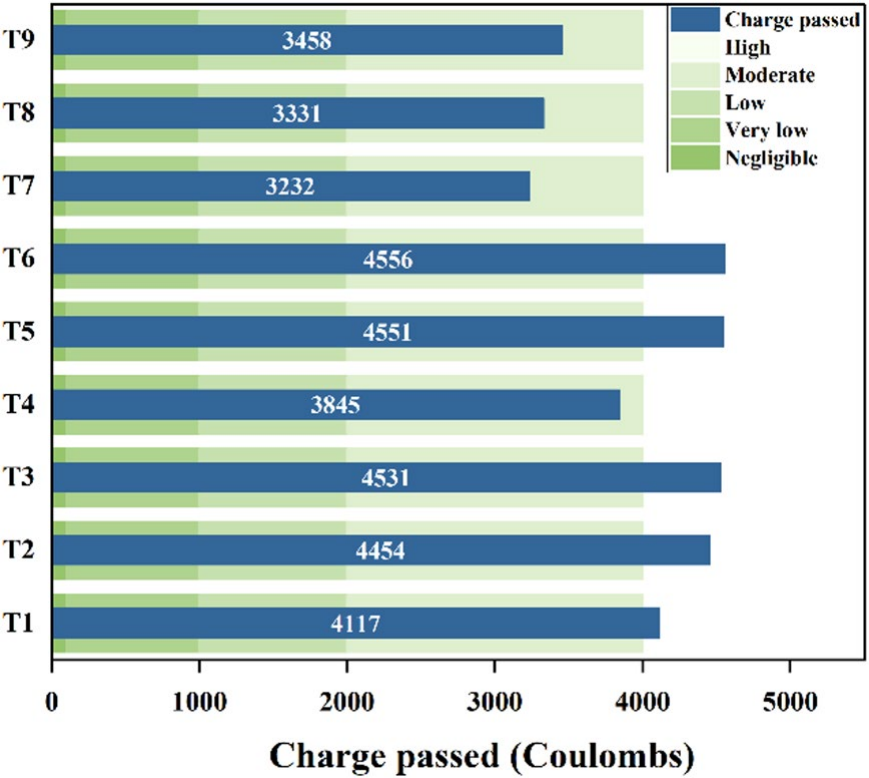
Chloride permeability results.

The chloride ion permeability was evaluated using the Rapid Chloride Permeability Test (RCPT) method as per ASTM C1202-12 ^71^ across all nine concrete mixes. Figure 20 presents the RCPT results for all nine mixes. Mixes incorporating higher proportions of GGBS exhibited a noticeable reduction in chloride ion permeability. Among the tested specimens, Mix T6 recorded the highest charge passed, indicating the greatest chloride ion permeability at 4556 coulombs. In contrast, Mix T7 demonstrated superior resistance to chloride ingress, with the lowest charge passed at 3232 coulombs.

**Fig. 21:**
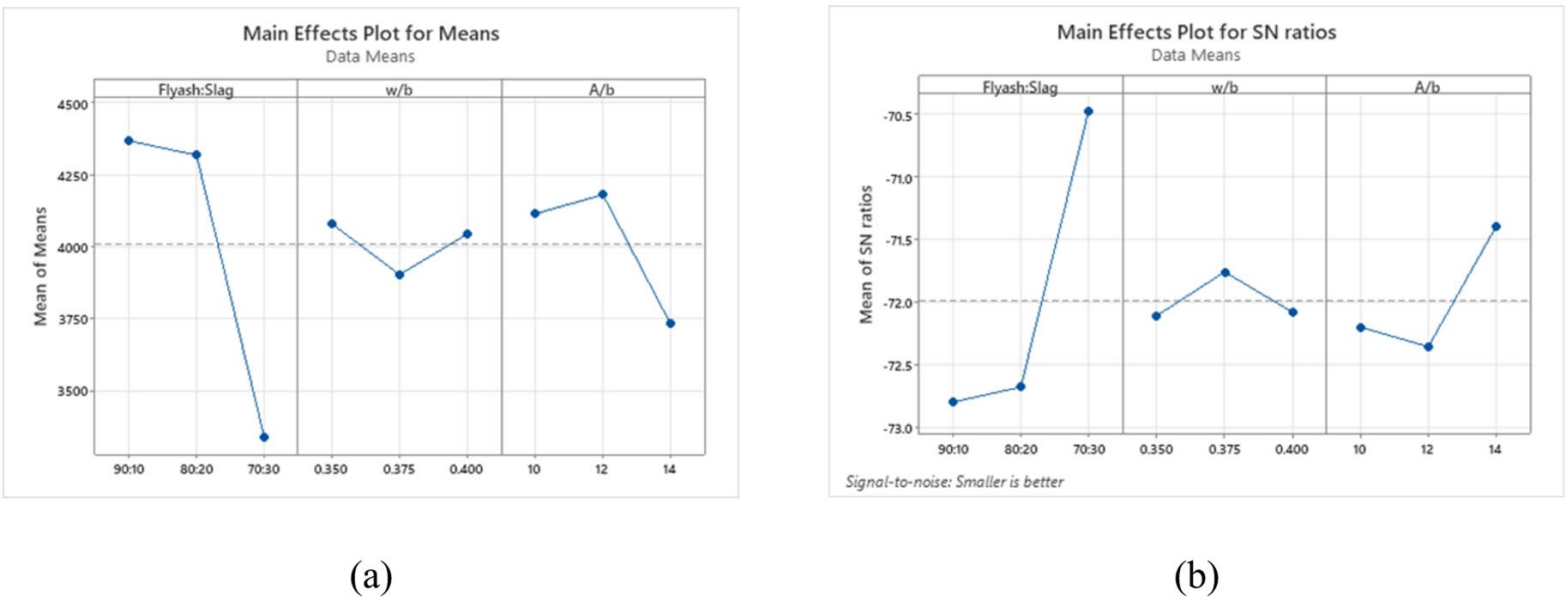
Main effects of (**a**) Means (**b**) S/N ratios of chloride permeability.

The chloride permeability analysis was conducted using the principle that lower S/N ratios correspond to improved performance, is shown in Fig. 21(a) and 21(b). The binder ratio most significantly affects chloride ion permeability, followed by w/b ratio and A/b ratio. A 70:30 binder ratio, 0.375 w/b ratio, and 14% A/b ratio showed minimal charge passed.

**Fig. 22:**
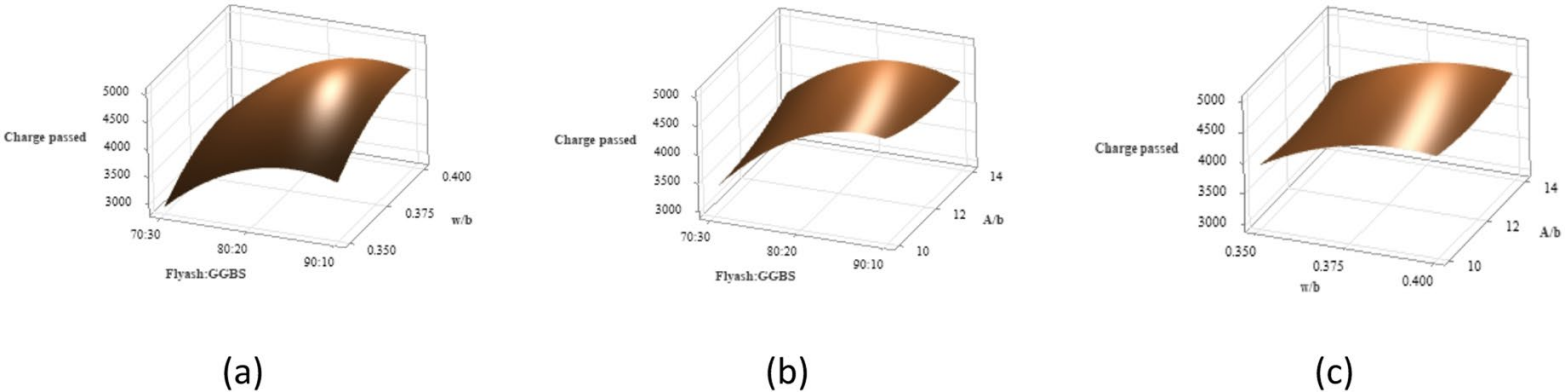
3D Surface plots for RCPT (**a**) Charge passed vs. Binder ratio, w/b. (**b**) Charge passed vs. Binder ratio, A/b (c) Charge passed vs. w/b, A/b.

Figure 22 displays 3D surface plots that illustrate the combined effects of different parameters on chloride permeability. In Fig. 22(a), it is evident that an increase in the w/b ratio leads to greater charge passage. This is primarily due to higher matrix porosity and enhanced capillary absorption, both promoting easier movement of chloride ions, as confirmed by RCPT outcomes. Among the tested mixes, the binder ratio 70:30 performed the best, recording the lowest charge passed and the highest compressive strength which can be linked to enhanced geopolymerization triggered by the calcium content in GGBS, which supports the formation of calcium-aluminosilicate-hydrate (C-A-S-H) gels. These gels help create a denser matrix, reduce pore connectivity, and significantly restrict ion transport^17^. In contrast, the 80:20 mix exhibited the highest charge passage, likely due to insufficient GGBS content, which impaired the continuity of the gel matrix and led to larger capillary pores, thereby increasing chloride ion permeability. While the 90:10 mix performed better than the 80:20 blend, its reduced GGBS content produced a less cohesive pore structure than the 70:30 binder ratio.

Figure 22(b) shows that increasing the FA content at a constant A/b ratio increases chloride ion permeability owing to the lower reactivity of FA compared to GGBS, which leads to a less dense and more porous microstructure, allowing easier ingress of chloride ions. However, excessive activator dosages may risk increasing porosity. In addition, high FA content coupled with increased activator dosage can lead to microcracks and discontinuous pores, ultimately degrading strength and durability.

Figure 22(c) shows that increasing A/b at a fixed w/b slightly raises chloride permeability due to higher capillary absorption. The 0.350 w/b mix had the lowest permeability and highest strength. Higher w/b ratios increased chloride ion permeability, with the highest at 0.400 w/b and 14% A/b due to porosity.

### Grey relational analysis

Grey Relational Analysis (GRA) evaluates multi-performance processes and identifies optimal solutions among interdependent variables. Data are normalized to a standard scale: the ‘larger-the-better’ criterion applies to slump, compressive, split tensile, and flexural strengths, while the ‘smaller-the-better’ criterion applies to sorptivity and RCPT via Eqs. 1 and 2.

(i) For “smaller the better,”
  1 $$\:\mathrm{Y}\mathrm{i}\mathrm{n}\:=\left|\frac{\mathrm{max}\left({Y}_{i\:}\right)\:-\:{Y}_{i}}{\mathrm{max}\left({Y}_{i\:}\right)\:-\mathrm{min}({Y}_{i})}\right|$$
(ii) For “larger the better,”
  2 $$\:\mathrm{Y}\mathrm{i}\mathrm{n}\:=\left|\frac{{Y}_{i}\:-\:\mathrm{min}\left({Y}_{i\:}\right)\:}{\mathrm{max}\left({Y}_{i\:}\right)\:-\mathrm{min}({Y}_{i})}\right|$$

In the subsequent phase of Grey Relational Analysis (GRA), the deviation sequence (Δ_i_) is determined, representing the difference between the normalized value of each trial and that of the optimal trial. This is computed using Eq. 3.

3 $$\:\mathrm{D}\mathrm{e}\mathrm{v}\mathrm{i}\mathrm{a}\mathrm{t}\mathrm{i}\mathrm{o}\mathrm{n}\:\mathrm{s}\mathrm{e}\mathrm{q}\mathrm{u}\mathrm{e}\mathrm{n}\mathrm{c}\mathrm{e},\:{\Delta\:}\mathrm{i}\:=\left|{Y}_{in}-\mathrm{max}\left({Y}_{in}\right)\right|$$

The next step involves calculating the Grey Relational Coefficient (GRC), denoted as ζ_i_, using Eq. 4, which includes a distinguishing coefficient (ξ) typically set to 0.5 and selected within the range [0,1]. After obtaining the GRCs for all responses, the Grey Relational Grade (GRG) is computed using Eq. 5. In this study, equal weighting was assigned to all responses (workability, strength, and durability), since no predefined priority was established among them. This approach ensures unbiased multi-response optimization. The GRG represents the weighted average of the corresponding GRCs and serves as an overall indicator of performance.

4 $$\:\mathrm{G}\mathrm{R}\mathrm{C},\:{\upzeta\:}\mathrm{i}\:=\frac{{\varDelta\:}_{min}+\:\xi\:*\:{\varDelta\:}_{max}}{{\varDelta\:}_{i}+\:\xi\:*\:{\varDelta\:}_{max}}$$

5 $$\:\mathrm{G}\mathrm{R}\mathrm{G},\:{\Upsilon\:}\mathrm{i}\:=\frac{1}{n}\:\sum\:_{k-1}^{n}{w}_{i}\:{\zeta\:}_{i}\left(k\right)$$

Considering all evaluated properties, Mix T7 demonstrated superior performance and achieved the highest rank, with a binder ratio of 70:30, a w/b ratio of 0.350, and an A/b ratio of 14%. The optimization process can be further refined by evaluating the GRG across all nine mix designs. Enhanced GRG scores are indicative of improved multi-response behavior of the mix. However, upon further analysis, the proposed optimal combination did not improve GRG. Therefore, the optimal mix identified in Table 5 is considered the most suitable for structural-grade AAC.

**Table 5:** Normalization, deviation sequences, grey relational coefficients, Grade, and Rank.

Mix ID	Normalization						Deviation sequence						Grey relational coefficient - GRC						Grade	Rank
	Slump	CS	TS	FS	Sorptivity	RCPT	Slump	CS	TS	FS	Sorptivity	RCPT	Slump	CS	TS	FS	Sorptivity	RCPT		
T1	0.000	0.101	0.042	0.071	0.400	0.332	1.000	0.899	0.958	0.929	0.600	0.668	0.333	0.357	0.343	0.350	0.455	0.428	0.378	9
T2	0.500	0.119	0.115	0.085	0.317	0.077	0.500	0.881	0.885	0.915	0.683	0.923	0.500	0.362	0.361	0.353	0.423	0.351	0.392	8
T3	1.000	0.000	0.000	0.000	0.450	0.019	0.000	1.000	1.000	1.000	0.550	0.981	1.000	0.333	0.333	0.333	0.476	0.338	0.469	7
T4	0.100	0.766	0.527	0.602	0.267	0.537	0.900	0.234	0.473	0.398	0.733	0.463	0.357	0.681	0.514	0.557	0.405	0.519	0.506	4
T5	0.400	0.552	0.442	0.580	0.617	0.004	0.600	0.448	0.558	0.420	0.383	0.996	0.455	0.528	0.473	0.543	0.566	0.334	0.483	5
T6	0.700	0.670	0.470	0.523	0.000	0.000	0.300	0.330	0.530	0.477	1.000	1.000	0.625	0.602	0.485	0.512	0.333	0.333	0.482	6
**T7**	**0.200**	**1.000**	**1.000**	**1.000**	**1.000**	**1.000**	**0.800**	**0.000**	**0.000**	**0.000**	**0.000**	**0.000**	**0.385**	**1.000**	**1.000**	**1.000**	**1.000**	**1.000**	**0.897**	**1**
T8	0.100	0.751	0.742	0.787	0.667	0.925	0.900	0.249	0.258	0.213	0.333	0.075	0.357	0.668	0.660	0.701	0.600	0.870	0.643	3
T9	0.600	0.680	0.700	0.864	0.667	0.829	0.400	0.320	0.300	0.136	0.333	0.171	0.556	0.610	0.625	0.786	0.600	0.745	0.654	2

### Microstructural analysis

#### SEM analysis

**Fig. 23:**
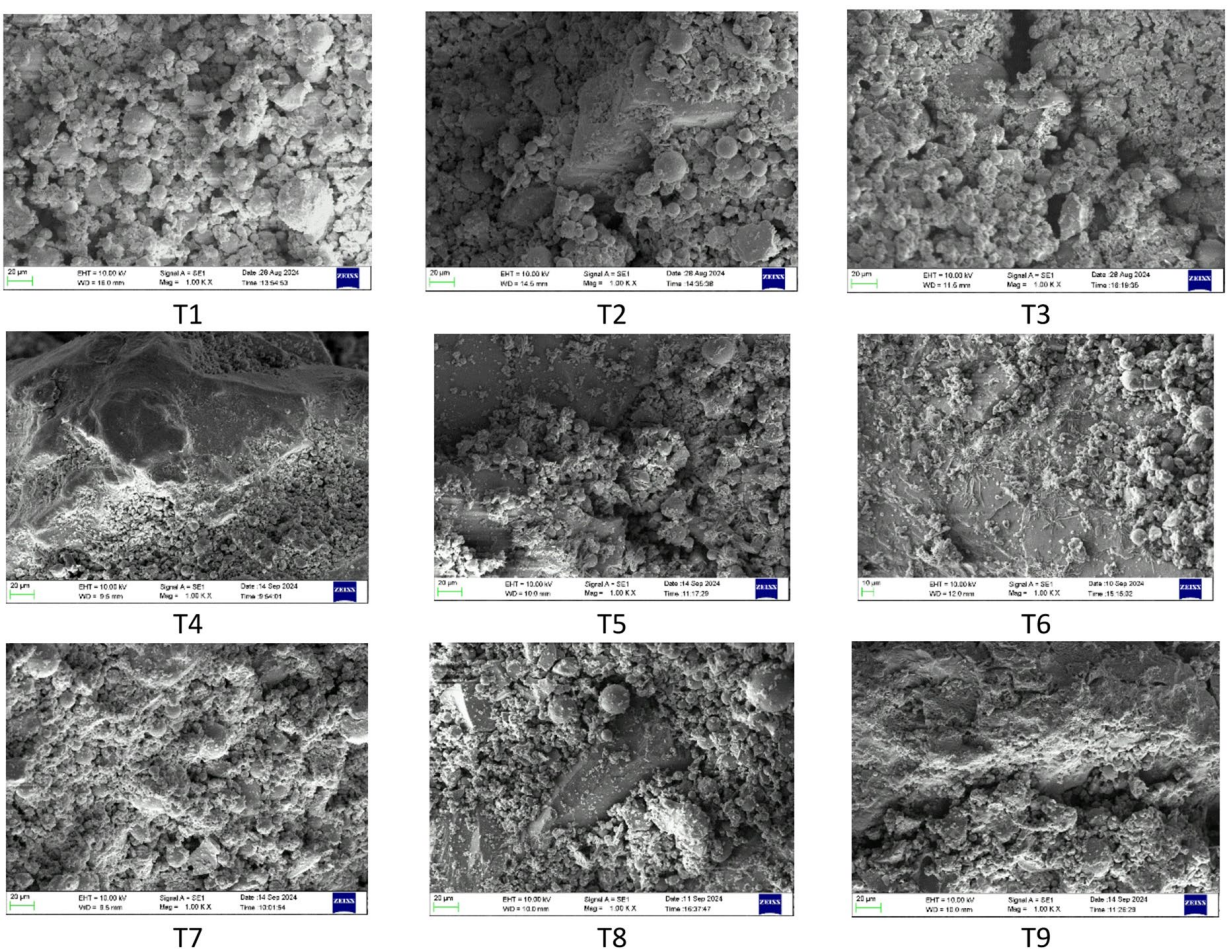
SEM images of all nine trial mixes.

The SEM images shown in Fig. 23 revealed a progressive improvement in microstructural development with increasing GGBS content and reduced w/b ratios and optimized activator dosage.

Low-GGBS mixes (T1–T3) exhibited a porous, weakly bonded matrix containing numerous unreacted FA spheres coated with a thin layer of amorphous N–A–S–H gel. The limited calcium availability restricted gel polymerization, resulting in a discontinuous binder phase and poor particle interlocking. This microstructural deficiency corresponds to the lower mechanical performance observed in these mixes.

In the 80:20 FA-GGBS mixes (T4–T6), the additional calcium supplied by GGBS promoted the formation of C-A-S-H and hybrid (N, C)-A-S-H gels, leading to a denser and more cohesive microstructure. SEM images showed reduced porosity, improved particle bonding, and enhanced gel continuity, indicating more efficient geopolymerization and better matrix integrity compared to the mixes with 90:10 FA-GGBS systems.

The GGBS-rich mixes (T7–T9) demonstrated the most refined and continuous microstructures, dominated by calcium-rich C-A-S-H and hybrid (N, C)-A-S-H gels. These mixes exhibited minimal unreacted residues, tightly packed pore structures, and strong interparticle bonding. Among them, T7 (70:30 FA: GGBS, w/b = 0.35, A/b = 14%) exhibited the densest and most homogeneous matrix, attributed to optimal calcium availability and sufficient alkalinity that triggered rapid precursor dissolution and extensive gel formation, resulting in improved mechanical and durability properties. Mixes T8 and T9 also displayed dense matrices, though marginal variations in activator dosage and water content led to slightly less compact structures.

Overall, the SEM findings confirm a strong correlation between microstructural densification and mechanical and durability performance. The transition from a porous, N-A-S-H-dominated matrix in low-calcium mixes to a dense hybrid (N, C)-A-S-H gel network in GGBS-rich compositions explains the improved structural performance at higher GGBS contents. Mix T7 emerges as the optimum composition due to its superior gel development, minimal porosity, and well-balanced geopolymerization kinetics.

### XRD analysis

**Fig. 24:**
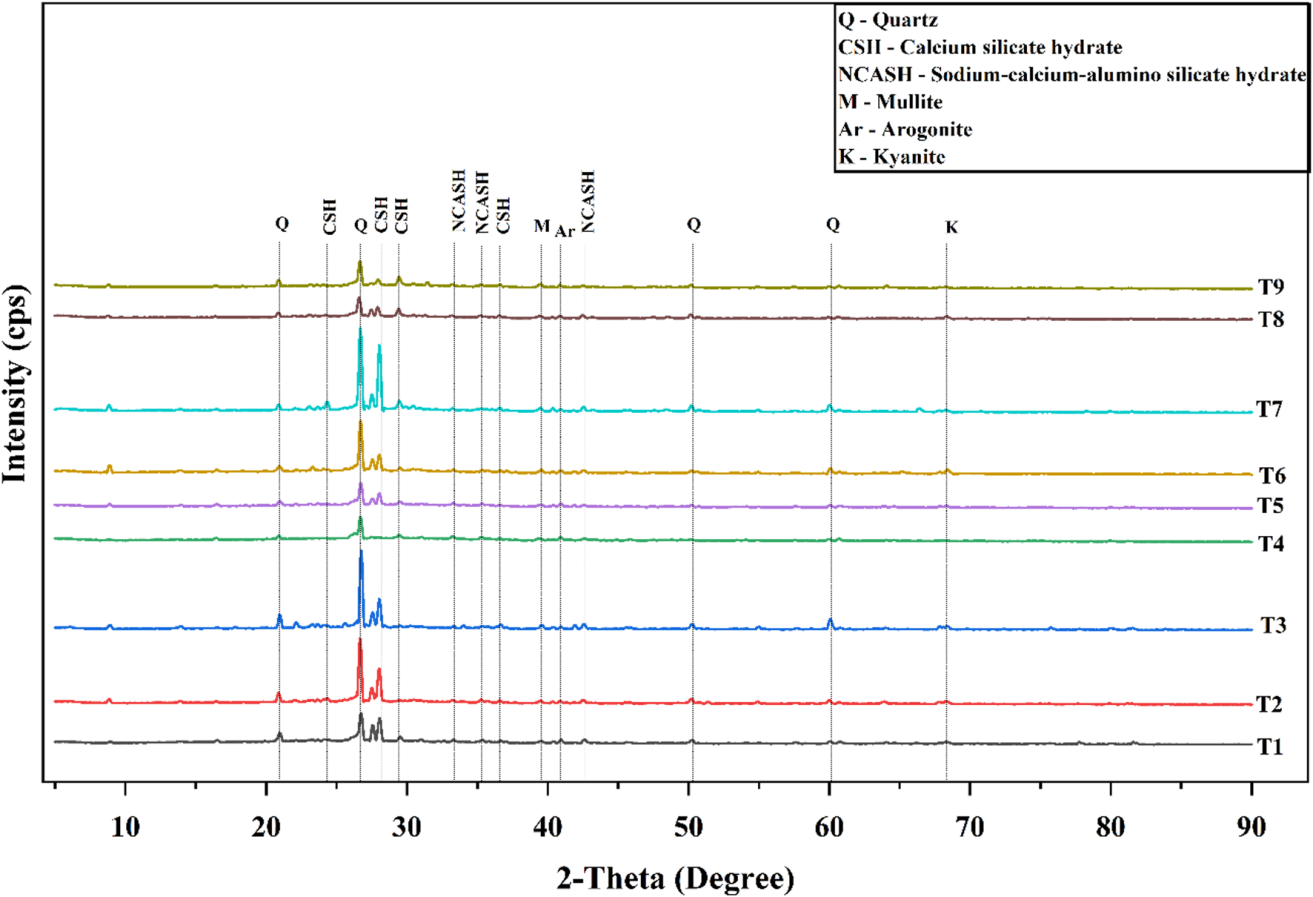
XRD patterns of all nine mixes.

The X-ray diffraction (XRD) patterns of the alkali-activated mixes after 28 days of curing (Fig. 24), scanned over a 2θ range of 5°−90°, reveal the progressive evolution of reaction products with varying GGBS content and activator dosage. A noticeable decrease in the intensity of quartz and mullite peaks, accompanied by the appearance of broad amorphous humps, indicates the advancement of geopolymerization and the formation of semi-crystalline gel phases. The development of new peaks attributable to hydration products further confirms ongoing chemical transformations responsible for mechanical strength enhancement.

Across all mixes, quartz (Q) remained the primary unreacted crystalline phase, with prominent peaks at 2θ ≈ 21.6°, 27.1°, and 50.4° (PDF: 00–005-0490). As the GGBS proportion and activator concentration increased, the diffraction peaks corresponding to calcium silicate hydrate (C-S-H) and sodium-calcium-alumino-silicate hydrate (N–C–A–S–H) became more pronounced, particularly in the ranges of 2θ ≈ 33°–36° and ≈ 43° (PDF: 00–003-0649, 00–025-0777). The enhanced intensity of these peaks signifies increased calcium participation, improved reactivity, and the formation of more stable, semi-crystalline binding gels. This observation aligns with previous findings reported by Sasui et al.^78^, Kamath et al^79^., Hamsashree et al^80^. and Gopal et al^81^..

These phase transformations indicate a clear calcium-enriched matrix consisting of C-A-S-H and N-C-A-S-H gels in GGBS-rich mixes. Among all compositions, Mix T7 showed the strongest C-S-H and N-C-A-S-H peaks, reflecting the highest degree of gel formation and matrix densification. This mineralogical profile correlates with its superior microstructure and highest compressive strength of 48.30 MPa, reinforcing its identification as the optimum mix for achieving maximum structural compactness and strength.

## Conclusions

- The OPAAC system demonstrates a sustainable and user-friendly alternative to traditional two-part systems by eliminating the need for handling corrosive liquid alkalis. This dry-mix, “just-add-water” approach simplifies on-site mixing, enhances worker safety, and offers greater potential for commercial scalability in the construction industry.
- The fresh performance of OPAAC was predominantly governed by the w/b ratio and A/b ratio and then the binder composition. Higher A/b ratios improved workability due to increased alkaline solution availability, whereas an elevated GGBS content reduced slump values owing to its angular morphology and high reactivity, which accelerated early setting.
- The compressive, tensile, and flexural strengths of OPAAC were significantly influenced by the binder ratio (FA to GGBS), then by w/b ratio and A/b ratio. Increased GGBS content contributed to enhanced mechanical strength through accelerated geopolymerization and dense matrix formation, while lower w/b ratios promoted improved strength due to reduced porosity and enhanced binder gel formation.
- Durability characteristics, evaluated through sorptivity and RCPT, were strongly influenced by the binder ratio, then by A/b ratio and w/b ratio. Mixes with higher GGBS content and lower w/b ratios exhibited reduced sorptivity and chloride ion permeability, attributable to a denser microstructure and lower capillary pore connectivity, enhancing resistance to fluid ingress.
- The Taguchi–Grey Relational Analysis (TiGRA) approach effectively identified the optimal mix parameters for maximizing workability, strength and durability. Among nine mixes, Mix T7 (70:30 FA: GGBS, w/b 0.35, A/b 14%) exhibited superior performance with M40 grade strength, low sorptivity, and minimal chloride permeability, confirming it as the best-performing formulation.
- SEM analysis revealed a dense and compact matrix in the mixes with higher GGBS content, indicating effective geopolymerization and reduced porosity. XRD results confirmed the presence of amorphous aluminosilicate gels along with crystalline phases such as C-S-H and N-C-A-S-H, validating the development of a stable and durable microstructure that correlates well with the mechanical and durability performance observed.
- Further research is recommended to evaluate the long-term durability performance of OPAAC under aggressive environmental conditions, including carbonation, sulphate attack, and freeze–thaw cycles. Additionally, scaling up OPAAC for structural applications, exploring the use of alternative industrial by-products as precursors, life-cycle assessment, and cost-benefit analyses can facilitate its adoption in mainstream construction practices.

### Practical implications

- A slump of approximately 150 mm is suitable for pumpable concrete, offering adequate flowability for efficient placement and ease of casting in reinforced sections; however, proper handling is required as field conditions such as temperature, moisture, and transport time can affect workability.
- The absolute volume method provides a simple and practical proportioning approach, ensuring consistent and reproducible OPAAC mixes by accounting for the true volume contribution of all constituents.
- High GGBS content in OPAAC increases sensitivity to activator dosage and accelerates workability loss due to rapid reaction of calcium-rich slag, indicating the need for careful control of temperature, mixing sequence, and curing practices in field applications.
- Mix combinations exhibiting very low strengths, especially those with high FA content and higher w/b ratios are impractical for structural use and should not be pursued further, especially in one-part systems under ambient curing conditions.

### Limitations

- The curing regime was kept constant for all mixes to focus on developing an ambient-curable FA-GGBS-based OPAAC. However, this limits understanding of how different curing conditions may affect performance.

#### Scope for future work

- Comparing the optimal mix (70:30 FA: GGBS, 0.35 w/b, 14% A/b) with conventional OPC concrete or two-part AAC would provide deeper insight into its practical competitiveness.
- Further investigation is needed to understand the factors contributing to extremely low strength in certain mixes (e.g., those with high FA or high w/b ratios) to refine formulation boundaries.
- Additional durability assessments such as carbonation resistance, freeze–thaw cycles, or sulphate attack should be included to provide a more comprehensive evaluation of OPAAC’s long-term performance.
- The occurrence of unreacted particles observed in microstructural analysis suggests a need to explore optimized activator concentrations or modified curing regimes to enhance geopolymerization efficiency.

## Data Availability

All data generated or analyzed during this study are included in this published article.
